# Children and Their Management in Disease

**Published:** 1884-06

**Authors:** T. J. Word

**Affiliations:** Atlanta, Ga.


### CHILDREN AND THEIR MANAGEMENT IN DISEASE.

BY T. J. WORD, M. D., ATLANTA, GA.

In submitting a few remarks on the following points connected with the diseases of children, and the ceremonial to be observed in our intercourse with them, I desire to say that they are offered in no self-important or vain-glorious spirit, but with an humble trust that such suggestions may not be needed by any considerable portion of the profession. The opinion or experience of any single member carries but little weight and is entitled to but little credence, until its merits have been settled by the crucial test of experience in a large number of instances, in different hands, with such an approximation to uniformity in the results as to leave the weight of evidence in its favor. In this way only can anything like certainty be attained in any department of the healing art, which, like the monument, is not constructed of a single stone, but stone by stone it rises to its cloud-capped pinnacle; or by single grains of experience, which, multiplied indefinitely, like the coral reef, swells from old ocean's depths and parts the living waters while formed of infinitesimal atoms combined into concrete whole upon which the angry waves, lashed to fury by the storm, recoil. So too must error, however popular, succumb when battling against a truth established and solidified out of these infinitesimal truths of individual experience combined into a concrete whole of finished proportions.

It is asserted by a distinguished writer (West) on the diseases of children that one-third of all our patients are to be found in the ranks of infancy and childhood. And notwithstanding this statement, the diseases of this numerous class have received less attention probably than any other branch of professional inquiry. This may to some extent be due to the conviction heretofore entertained by many of the profession, that anything like certainty could not be attained in diagnosing the diseases of children, and which finds expression in the remark that the diseases of infancy are either a *terra incognita*, not to be attempted, or else, in the more complacent declaration, that the diseases of the nursery belong to the domain especially of the old woman with her appliances of *sheep saffron* tea, *yarbs*, and other decoctions in which it is all guess work anyhow, and that these harmless expedients are just as likely to secure the desired results and are much less potent for injury, than the best directed efforts and resources of the most skillful physician. Such conclusions are by no means creditable in this enlightened age, and it is gratifying to note the fact that a better and more advanced opinion is gradually winning its way to professional favor, while these self-imposed impediments do not so frequently hinder or close the door to an earnest and patient inquiry in this direction. It is to be hoped such convictions will soon find no abiding place and be remembered with the absurdities of the past, and no longer embarrass this important branch of the healing art.

The same painstaking investigation bestowed in other lines cannot fail to yield a rich harvest from a proper acquaintance with the sign language of the diseases of children, and elevate it to its true place in both professional and popular estimation. The light shed upon it by the worthy men who have dignified it with their labors, has dispelled much of the gloom and darkness which so long overshadowed it, and have established landmarks and guides to a better acquaintance and higher achievement in the means for understanding and combating the diseases of infancy and childhood. To such as engage in this important department and pursue it to its legitimate results, maternal gratitude and benedictions will prove their highest award and sweetest praise.

The difficulties to be overcome in our ministrations among the little ones are often almost insurmountable, and are in many instances largely due to the thoughtlessness of mothers, or the wickedness of nurses, in using the doctor as the switch to secure obedience to their whims, and thus they instill into the minds of the children a dread of the doctor and cause them to regard him as a monster in human shape, whom they learn to fear and hate with equal intensity. Thus armed they naturally interpret his visit in no complacent spirit and construe his attentions as meaning only injury or mischief, and to be resisted by every device to the bitter end, and often with a success truly appalling, in closing every avenue for gaining an intelligent opinion of the disease, founded as it must be upon a right interpretation of the sign language, which only gives correct indications when the little patient is calm and unmoved by either fright or anger. How important, then, that these signals of distress should be properly displayed, as well as correctly interpreted, in guiding our conclusions. Could I speak a word of warning to every mother, it would be this: Never under any circumstances use the physician or his office in an unpleasant sense to her child, nor suffer it to be done, but on the contrary instill into the mind of her babe a love for the family physician, and encourage it to regard him as its best friend to whom it can go with its most familiar secrets and troubles, assured of his loving sympathy and willingness to relieve it of its real as well as imaginary troubles. Such a course would facilitate the investigations necessary to a correct diagnosis and a successful contest with the disease; would expedite the cure, multiply the triumphs of professional skill, and save many a mother from a desolate heart and an empty crib

In this connection it may not be amiss to mention that the physician plays an important part in making himself agreeable to children. A love for children will supply the needed instructions on this head. In the language of Scripture, he should become as a little child, especially in the sick room, in which such juvenile manifestations will propitiate their confidence and invite their cheerful submission to a familiar inspection and study of their troubles. In the next place, when visiting sick children the physician should leave behind him all hurry and bluster and enter the sick chamber in a gentle, quiet manner, with as little ceremony as possible, especially if it be a first visit to a strange child. In furtherance of this, and in order to allay any agitation caused by his presence, he should avoid all and every act calculated to awaken the child's apprehension that the visit is personal to itself, and to this end he should pay especial attention to the other and well children, should there be any, and while thus making himself agreeable to them by the many little arts and familiarities calculated to awaken their unreserved and playful fondness, he is also ingratiating himself into the good opinion of the sick one, and paving the way for an easy and unembarrassed inspection of the case. While thus engaged and listening to the history of the case as detailed by the mother or nurse, he may casually glance at the sick one, note its expression, position, or any exclamations or other evidences of suffering. The statement of the mother should always be patiently listened to; she of all others has the keenest detection of any and all evidences of sickness or departures from health in her babe, whether manifested in its general condition or expressed in the attitude, actions, temper, etc., cannot escape her maternal intuition. Therefore let her talk, however rambling and prolix her account may be; now and then a ray of light may be shed upon some important indication which will assist in directing your investigations into the proper channel. And then, patience with her in the recital has another advantage. She estimates your capacity very largely by the respect you manifest to her statements and conclusions, and thus while you are accustoming the child to your presence, you are ingratiating yourself into the mother's confidence and expediting rather than hindering the consummation of your visit. When we find the child sufficiently reconciled to our presence to begin a personal inspection, the question naturally presents itself how and in what manner shall I begin my inquiries? Each one's good sense and tact must be their guide in the explorations necessary to locate the trouble. When this has been done the next inquiry will be as to its nature—whether it be functional, sympathetic or organic. In some instances these questions are difficult to solve, and can only be done by a double examination, that is, to inspect the patient when asleep as well as when awake. In this way a correct diagnosis can generally be reached. Thus we see another indication for the exercise of patience which, in the language of Scripture, is to let this rare virtue "have its perfect work." In no other department will the practitioner find such constant demands for this invaluable commodity as in the study and investigation of the diseases of childhood; and for fear of putting that of my readers to an unbearable strain I will close this communication without entering further into the details of special indications furnished by disease in the various organs and structures of infantile economy.

TO BE CONTINUED.

